# Molecular diagnosis of hereditary spherocytosis by multi-gene target sequencing in Korea: matching with osmotic fragility test and presence of spherocyte

**DOI:** 10.1186/s13023-019-1070-0

**Published:** 2019-05-23

**Authors:** Hyoung Soo Choi, Qute Choi, Jung-Ah Kim, Kyong Ok Im, Si Nae Park, Yoomi Park, Hee Young Shin, Hyoung Jin Kang, Hoon Kook, Seon Young Kim, Soo-Jeong Kim, Inho Kim, Ji Yoon Kim, Hawk Kim, Kyung Duk Park, Kyung Bae Park, Meerim Park, Sang Kyu Park, Eun Sil Park, Jeong-A Park, Jun Eun Park, Ji Kyoung Park, Hee Jo Baek, Jeong Ho Seo, Ye Jee Shim, Hyo Seop Ahn, Keon Hee Yoo, Hoi Soo Yoon, Young-Woong Won, Kun Soo Lee, Kwang Chul Lee, Mee Jeong Lee, Sun Ah. Lee, Jun Ah Lee, Jae Min Lee, Jae Hee Lee, Ji Won Lee, Young Tak Lim, Hyun Joo Jung, Hee Won Chueh, Eun Jin Choi, Hye Lim Jung, Ju Han Kim, Dong Soon Lee, The Hereditary Hemolytic Anemia Working Party of the Korean Society of Hematology

**Affiliations:** 1 Department of Pediatrics, Seoul National University Bundang Hospital, Seongnam, Republic of Korea; 2 Department of Laboratory Medicine, Chungnam National University Hospital, Daejeon, Republic of Korea; 3 Department of Laboratory Medicine, Seoul National University College of Medicine, 101, Daehak-ro, Jongno-gu, Seoul, 03080 Republic of Korea; 4 Cancer Research Institute, Seoul National University College of Medicine, Seoul, Republic of Korea; 5 Division of Biomedical Informatics, Seoul National University Biomedical Informatics (SNUBI), Seoul National University College of Medicine, 101, Daehak-ro, Jongno-gu, Seoul, 03080 Republic of Korea; 6 Department of Pediatrics, Seoul National University College of Medicine, Seoul, Republic of Korea; 7 Department of Pediatrics, Chonnam National University Hwasun Hospital, Chonnam National University Medical School, Gwangju, Republic of Korea; 8 Department of Laboratory Medicine, Chungnam National University School of Medicine, Daejeon, Republic of Korea; 9 Division of Hematology, Department of Internal Medicine, Yonsei University College of Medicine, Severance Hospital, Seoul, Republic of Korea; 10 Department of Internal Medicine, Seoul National University College Medicine, Seoul, Republic of Korea; 11 Department of Pediatrics, Kyungpook National University School of Medicine, Daegu, Republic of Korea; 12 Department of Hematology and Oncology, Ulsan University Hospital, University of Ulsan College of Medicine, Ulsan, Republic of Korea; 13 Department of Pediatrics, Soonchunhyang University Hospital Cheonan, Cheonan, Republic of Korea; 14 Department of Pediatrics, Chungbuk National University College of Medicine, Cheongju, Republic of Korea; 15 Department of Pediatrics, Ulsan University Hospital, Ulsan, Republic of Korea; 16 Department of Pediatrics, Gyeongsang National University College of Medicine, Jinju, Republic of Korea; 17 Department of Pediatrics, Inje University College of Medicine, Busan, Republic of Korea; 18 Department of Pediatrics, Ajou University School of Medicine, Suwon, Republic of Korea; 19 Department of pediatrics, Inje University College of Medicine, Busan Paik Hospital, Busan, Republic of Korea; 20 Department of Pediatrics, Pusan National University College of Medicine, Yangsan, Republic of Korea; 21 Department of Pediatrics, Keimyung University School of Medicine and Dongsan Medical Center, Daegu, Republic of Korea; 22 Department of Pediatrics, Sungkyunkwan University School of Medicine, Samsung Medical Center, Seoul, Republic of Korea; 23 Department of Pediatrics, Kyung Hee University School of Medicine, Seoul, Republic of Korea; 24 Department of Internal Medicine, Hanyang University Guri Hospital, Guri, Republic of Korea; 25 Department of Pediatrics, Korea University College of Medicine, Seoul, Republic of Korea; 26 Department of Pediatrics, University of Dankook College of Medicine, Cheonan, Republic of Korea; 27 Department of Internal Medicine, Daegu Fatima Hospital, Daegu, Republic of Korea; 28 Department of Pediatrics, Korea Cancer Center Hospital, Seoul, Republic of Korea; 29 Department of Pediatrics, College of Medicine, Yeungnam University, Daegu, Republic of Korea; 30 Department of Pediatrics, Chosun University School of Medicine, Gwangju, Republic of Korea; 31 Department of Pediatrics, Dong-A University College of Medicine, Busan, Republic of Korea; 32 Department of Pediatrics, Daegu Catholic University, Daegu, Republic of Korea; 33 Department of Pediatrics, Sungkyunkwan University School of Medicine, Seoul, Republic of Korea; 34 The Korean Society of Hematology, Seoul, Republic of Korea

**Keywords:** Hereditary spherocytosis, RBC membrane disorder, Molecular diagnosis

## Abstract

**Background:**

Current diagnostic tests for hereditary spherocytosis (HS) focus on the detection of hemolysis or indirectly assessing defects of membrane protein, whereas direct methods to detect protein defects are complicated and difficult to implement. In the present study, we investigated the patterns of genetic variation associated with HS among patients clinically diagnosed with HS.

**Methods:**

Multi-gene targeted sequencing of 43 genes (17 RBC membrane protein-encoding genes, 20 RBC enzyme-encoding genes, and six additional genes for the differential diagnosis) was performed using the Illumina HiSeq platform.

**Results:**

Among 59 patients with HS, 50 (84.7%) had one or more significant variants in a RBC membrane protein-encoding genes. A total of 54 significant variants including 46 novel mutations were detected in six RBC membrane protein-encoding genes, with the highest number of variants found in *SPTB* (*n* = 28), and followed by *ANK1* (*n* = 19), *SLC4A1* (*n* = 3), *SPTA1* (*n* = 2), *EPB41* (*n* = 1), and *EPB42* (*n* = 1). Concurrent mutations of genes encoding RBC enzymes (*ALDOB, GAPDH,* and *GSR*) were detected in three patients. *UGT1A1* mutations were present in 24 patients (40.7%). Positive rate of osmotic fragility test was 86.8% among patients harboring HS-related gene mutations.

**Conclusions:**

This constitutes the first large-scaled genetic study of Korean patients with HS. We demonstrated that multi-gene target sequencing is sensitive and feasible that can be used as a powerful tool for diagnosing HS. Considering the discrepancies of clinical and molecular diagnoses of HS, our findings suggest that molecular genetic analysis is required for accurate diagnosis of HS.

**Electronic supplementary material:**

The online version of this article (10.1186/s13023-019-1070-0) contains supplementary material, which is available to authorized users.

## Background

Hereditary spherocytosis (HS) is the most common cause of hereditary hemolytic anemia (HHA) characterized by the presence of spherocytes in peripheral blood smear (PBS) [1, 2]. HS occurs in 1 in 2000 Caucasians, with less common frequency in Asians [1, 3, 4]. The crude incidence of HS in Korea was reported as 1 in every 5000 births [5]. Approximately 75% cases of HS are inherited as autosomal dominant (AD) mutations, whereas the remaining cases involve autosomal recessive (AR) or *de-novo* mutations [1].

HS is caused by a deficiency in or dysfunction of membrane proteins, including spectrin, ankyrin 1, band 3, and protein 4.2, associated with the RBC cytoskeleton [3, 4, 6]. Defective membrane proteins disrupt the vertical linkage between the RBC membrane cytoskeleton and the phospholipid bilayer, causing RBCs to lose its biconcave characteristics and become spherical in shape [3, 4, 6]. This abnormal RBC morphology leads to osmotically fragile cells that are selectively trapped and destroyed in the spleen [3, 4, 6]. A major clinical manifestation of HS is hemolytic anemia, which exhibits a wide range of clinical manifestations from asymptomatic to life-threatening anemia requiring regular RBC transfusions [1, 2]. Other clinical symptoms include splenomegaly, jaundice, and gallstones, depending on disease severity [1, 2].

**Fig. 1:**
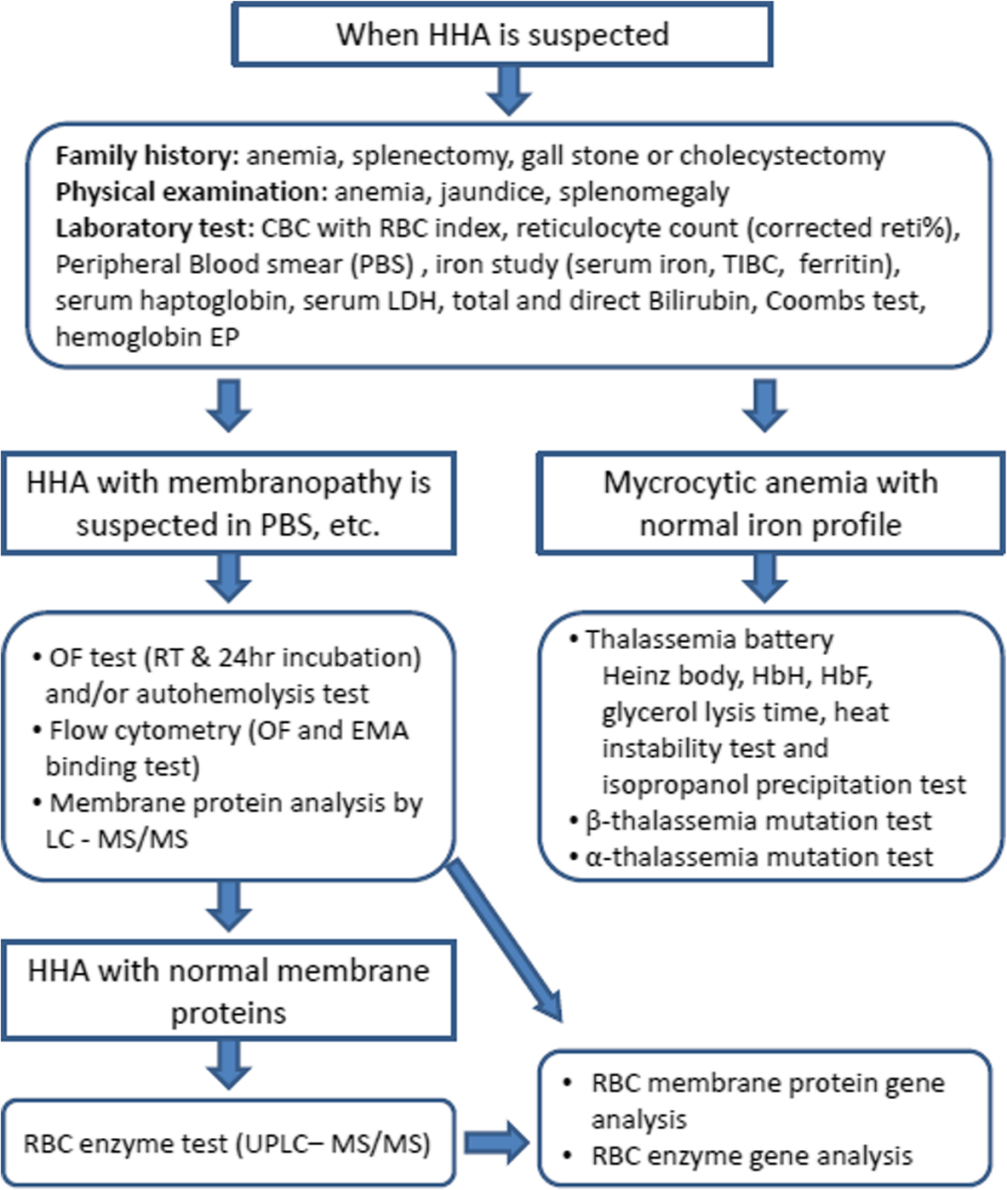
Standard operating procedure for the diagnosis of hereditary hemolytic anemia (HHA) by HHA Working Party of Korean Society of Hematology [5]

We have been operating the Korean Hereditary Hemolytic Anemia Working Party (KHHAWP) of the Korean Society of Hematology for 7 years since 2010, which name has been changed to RBC Disorder Working Party since November 2016. From 2007 to 2011, 195 patients (121 males and 74 females) diagnosed with HHA from 25 institutions were registered [7]. The KHHAWP presented standard operating procedure (SOP) for the diagnosis of HHA (Fig. 1) [5], which is similar to ICSH (International Council for Standardization in Haematology) guideline [8] except for excluding acid glycerol lysis time test as a screening test. Instead of gel electrophoresis analysis of erythrocyte membranes, the KHHAWP adopted mass spectrometry method as a confirmatory test, which is performed in one central laboratory in Korea.

The diagnosis of HS is based upon a combination of positive family history, clinical features and presence of spherocytes in PBS, which are detectable in 97% of patients [9]. When the diagnosis of HS is equivocal, additional laboratory tests are recommended such as osmotic fragility test (OFT), autohemolysis test, flow cytometry [OFT and eosin-5-maleimide (EMA) binding test] for screening test, and protein analysis using gel electrophoresis or mass spectrometry can be additionally tested [10–16]. However, none of the current diagnostic test can detect all patients with HS.

Considering the limitations of existing diagnostic tests, development of a simple and direct method to measure RBC membrane protein abnormalities to confirm HS is required. Analysis of RBC membrane protein-encoding genes is expected that it can be used complementarily with the conventional confirmatory tests [1, 11]. Multi-gene target sequencing for RBC membrane protein-encoding genes is feasible and reliable diagnostic method to detect mutations in patients affected by various disorders of the RBC membrane. Particularly, gene testing is important in young children with congenital anemia, transfusion-dependent patients, and in families with variable clinical expression or complex inheritance patterns [17–19].

In the present study, we investigated the genetic variation of RBC membrane protein-encoding genes using multi-gene target sequencing, comparing with clinical features. A total of 43 genes was included; 17 RBC membrane protein-encoding genes and 20 RBC enzyme-encoding genes, in context with six additional candidate genes for the purpose of differential diagnoses [thalassemia, congenital dyserythropoietic anemia (CDA), paroxysmal nocturnal hemoglobinuria (PNH), and Gilbert syndrome].

## Methods

### Patients

A total of 59 patients with HS including 31 males and 28 females with a median age of 7 years (range: 1–81 years), were registered between July 2013 and July 2014 from the pediatrics and internal medicine departments of 25 institutions in Korea. HS was diagnosed according to the SOP recommended by the KHHAWP of the Korean Society of Hematology (Fig. 1) [5].

Along with clinical data including age, sex, symptoms and family history, we collected the results of laboratory tests including CBC with RBC index, reticulocyte count, total and direct bilirubin concentration, lactate dehydrogenase (LDH), iron, total iron-binding capacity (TIBC), ferritin, PBS, and OFT by reviewing medical records (Table 1). Blood samples were collected from each patient after obtaining their written consent.

**Table 1:** Clinical characteristics of patients with HS in Korea

Characteristics	Total patients (*n* = 59)	Patients with gene mutation (*n* = 50)	Patients without gene mutation (*n* = 9)	*P* value between group with mutation vs. without mutation
Sex, *n* (%)				0.597
Male	31 (52.0)	27 (54.0)	4 (44.4)	
Female	28 (48.0)	23 (46.0)	5 (55.6)	
Age (years)				0.566
Median	7	7	8	
Range	1–81	1–81	2–17	
Family history of HS, *n* (%)				0.139
Positive	20 (33.9)	16 (32.0)	4 (44.4)	
Negative	39 (66.1)	34 (68.0)	5 (55.6)	
Clinical symptoms, *n* (%)				
Splenomegaly	38/59 (64.4)	31/50 (62.0)	7/9 (77.8)	0.363
Neontal jaundice	28/54 (51.9)	24/45 (53.3)	4/9 (44.4)	0.724
Hepatomegaly	9/53 (17.0)	9/44 (20.5)	1/9 (11.1)	1.000
Splenectomy	13/58 (22.4)	10/49 (20.4)	3/9 (40.0)	0.398
Aplastic crisis	14/56 (25.0)	11/47 (23.4)	3/9 (30.0)	0.676
Gallstones	10/57 (17.5)	9/48 (18.8)	1/9 (33.3)	1.000
Hematologic parameters, mean				
Hemoglobin (g/dL) (range)	8.4 (3.6–13.6)	8.4 (3.6–13.6)	8.3 (5.8–12.1)	0.476
MCV (fL) (range)	80.9 (62.3–107.0)	80.6 (62.3–107.0)	85.3 (70.4–107.0)	0.209
MCHC (g/dL) (range)	35.3 (30.8–38.2)	35.2 (30.8–38.2)	35.2 (31.5–37.9)	0.279
Markers of hemolysis, mean				
Reticulocyte count (%) (range)	7.5 (0.5–24.8)	7.4 (0.5–24.8)	7.2 (3.4–13.3)	0.461
Total bilirubin (mg/dL) (range)	4.1 (0.8–19.1)	4.0 (0.8–19.1)	4.3 (1.1–6.4)	0.320
Direct bilirubin (mg/dL) (range)	0.7 (0.2–1.3)	0.7 (0.3–1.3)	0.6 (0.4–0.8)	0.640
LDH (IU/L) (range)	508 (187–1557)	522 (187–1557)	448 (198–737)	0.843
Iron status parameters, mean				
Iron (μr/dL) (range)	101 (26–245)	98 (26–159)	111 (51–245)	0.198
TIBC (μT/dL) (range)	266 (108–486)	269 (108–486)	241 (195–274)	0.769
Ferritin (ng/mL) (range)	342 (32–4671)	360 (32–4671)	339 (74–278)	0.657
Grading of peripheral spherocytes, *n* (%)				0.622
0	5 (8.5)	4 (8.0)	1 (11.1)	
1+ or slight (2–5%),	18 (30.5)	15 (30.0)	3 (33.3)	
2+ or moderate (6–15%),	20 (33.9)	16 (32.0)	4 (44.4)	
3+ or marked (> 16%)	16 (27.1)	15 (30.0)	1 (11.1)	
Sex, *n* (%)				0.597
Male	31 (52.0)	27 (54.0)	4 (44.4)	
Female	28 (48.0)	23 (46.0)	5 (55.6)	
Severity, *n* (%)				0.678
Mild	6 (10.2)	5 (10.0)	1 (11.1)	
Moderate	27 (45.8)	24 (48.0)	3 (33.3)	
Severe	26 (44.1)	21 (42.0)	5 (55.6)	
Osmotic fragility tests, *n* (%)				0.614
Positive	41 (69.5)	33 (66.0)	8 (88.9)	
Negative	6 (10.2)	5 (10.0)	1 (11.1)	
NA	12 (20.3)	12 (24.0)	0	

Abbreviation: *HS* hereditary spherocytosis, *NA* not assessable

### Targeted sequencing

To gain insight into the genetic variations, we performed targeted sequencing for 43 gene panel (Additional file 1: Table S1). gDNA shearing to generate the standard library and the hybridization step targeting only exonic regions were performed by Celemics Inc. (Seoul, Korea). The final quality was assessed using the Agilent 2200 TapeStation System (Santa Clara, CA, USA). We sequenced a total target length of 259-kb regions using the paired-end 150-bp rapid-run sequencing mode on an Illumina HiSeq 2500 platform. The mean sequencing depth for the targeted regions (259-kb) was 231-fold (*n* = 59). Because a matched control sample was not included in this study, we applied a stringent variant selection pipeline to prioritize the high-confidence set of somatic mutations.

### Variant calling

The filtration process was performed as follows. Variants within non-exonic regions were removed. Variants that do not have enough depth were also filtered out to remove false positives. Common variants on 1000 genome projects with more than 5% of allele frequency were filtered out. CADD score shows predictive pathogenicity of variants. It considers diverse annotations from allelic diversity to functionality, in order to estimate pathogenic variants. In this study, CADD scores below 10 were cut-off for filtration. After these filters, in-house variants were also removed to make filtered variant lists. Validation of variant call was performed by target gene sequencing of involved genes.

### Simulation of the effect of mutated genes on protein structure

To predict how gene mutation affect protein structure, we visualized three-dimensional (3-D) spatial protein structure following acquisition of their structural information (http://www.proteinmodelportal.org) (Additional file 1: Table S2). We used PyMOL (http://www.pymol.org) to visualize 3-D representations of the protein, modified protein structures based on genetic mutation profiles from next-generation sequencing (NGS) results.

### Statistical analyses

Stata/SE (v.14; StataCorp, College Station, TX, USA) was used for data analyses. Statistical differences in terms of continuous clinical characteristic variables were estimated by two sample *t* test. The significance of differences in categorical variables between groups was determined by the Pearson χ2 test or Fisher’s exact test. The level of significance was set at *P* < 0.05.

## Results

### Clinical characteristics

Among 59 patients with HS, 20 (33.9%) had a family history of HS, whereas symptoms of splenomegaly, neonatal jaundice, and hepatomegaly were exhibited in 38 of 59 (64.4%), 28 of 54 (51.9%), and 10 of 59 (16.7%) patients, respectively. Mean values for laboratory tests were as follows: hemoglobin concentration 8.4 g/dL (3.6–13.6 g/dL); corpuscular volume 80.9 fL (62.3–107.0 fL); corpuscular hemoglobin concentration 35.3 g/dL (30.8–38.2 g/dL); reticulocyte count indicating hemolysis 7.5% (0.5–24.8%); total bilirubin/direct bilirubin 4.1/0.7 mg/dL (0.8–19.1/0.2–1.3 mg/dL); LDH 508 IU/L (187–1557 IU/L); parameters representing iron profile, including iron 101 μg/dL (26–245 μg/dL), TIBC 266 μg/dL (108–486 μg/dL); and ferritin concentration, 342 ng/mL (32–4671 ng/mL). PBS was rated for spherocytes on a four-point scale [20] from 0, 1+ or slight (2–5%), 2+ or moderate (6–15%), and 3+ or marked (> 16%) and the number of smears returning 0, 1+ or slight, 2+ or moderate and 3+ or marked were 5 (8.5%), 18 (30.5%), 20 (33.9%), and 16 (27.1%) patients, respectively. According to HS-severity criteria [11], severe, moderate, and mild cases were 26 (44.1%), 27 (45.8%), and 6 (10.2%) patients, respectively (Table 1).

### Variants profile of RBC membrane protein-encoding genes

**Fig. 2:**
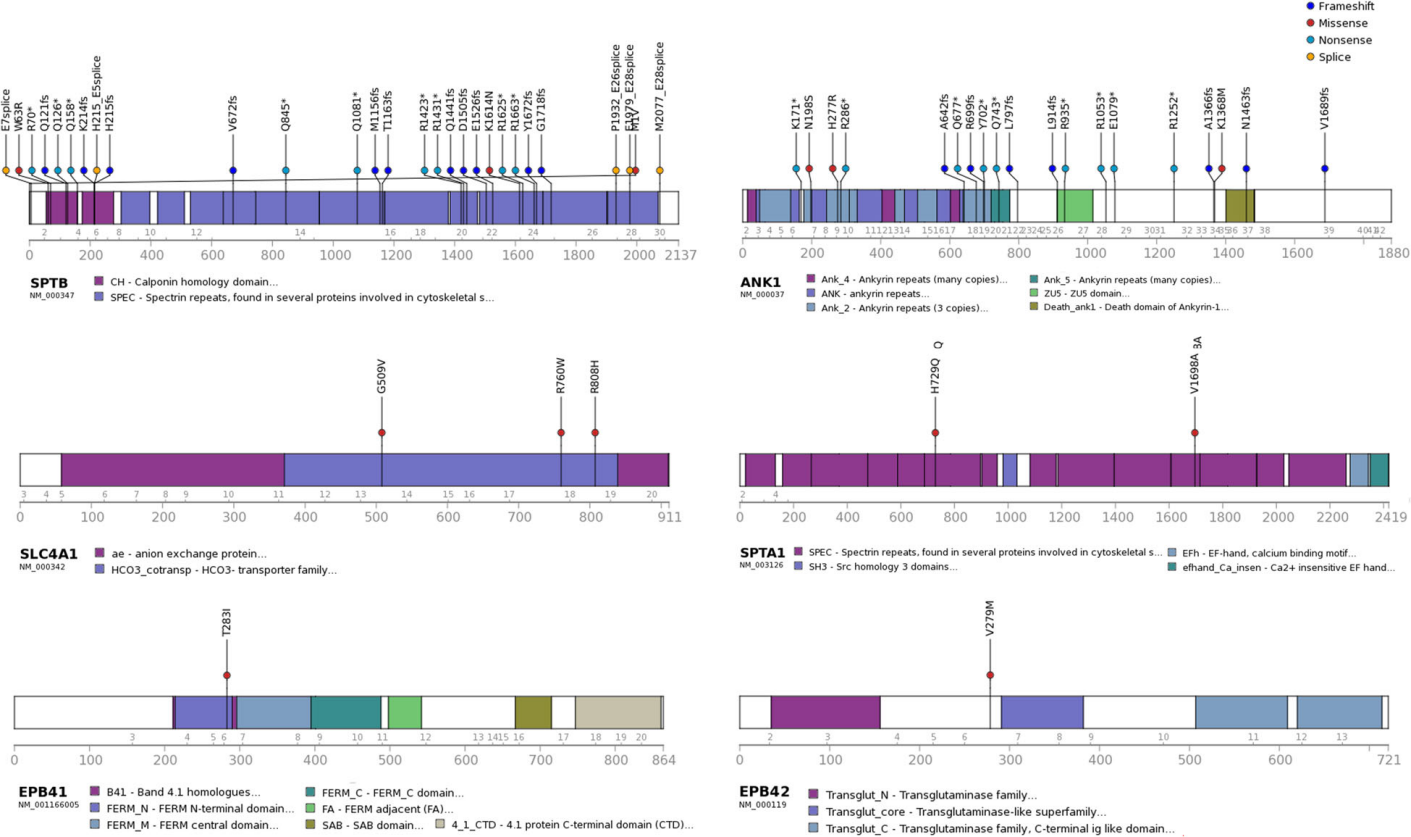
Characteristics of significant variants for RBC membrane protein-encoding genes; *SPTB*, *ANK1*, *SLC4A1*, *SPTA1*, *EPB41*, *EPB42*. Abbreviations: *SPTB,* spectrin, beta; *ANK1,* ankyrin 1; *SLC4A1,* solute carrier family 4, member 1; *SPTA1,* spectrin, alpha 1; *EPB41,* erythrocyte membrane protein band 4.1; *EPB42,* erythrocyte membrane protein band 4.2

Among 17 RBC membrane protein-encoding genes examined, significant disease-related mutations were observed in six: *SPTB* (spectrin, beta), *ANK1* (ankyrin 1), *SLC4A1* (solute carrier family 4, member 1), *SPTA1* (spectrin, alpha 1), *EPB41* (erythrocyte membrane protein band 4.1), and *EPB42* (erythrocyte membrane protein band 4.2) (Fig. 2). A total of 54 significant mutations were observed, of which eight were previously reported as pathogenic in patients with HS and 46 variants were novel mutations (Additional file 1: Table S3). The highest number of mutations were found in *SPTB* (*n* = 28), and followed by *ANK1* (*n* = 19), *SLC4A1* (*n* = 3), *SPTA1* (*n* = 2), *EPB41* (*n* = 1), and *EPB42* (*n* = 1). According to the American College of Medical Genetics and Genomics guidelines [21], 12 were pathogenic mutations (including eight previously reported variants), 29 were likely pathogenic mutations, and 13 were classified as having uncertain significance. All the variants have been confirmed by Sanger sequencing using 35 primer sets (Additional file 1: Table S4).

### Variant characteristics in patients with HS

**Fig. 3:**
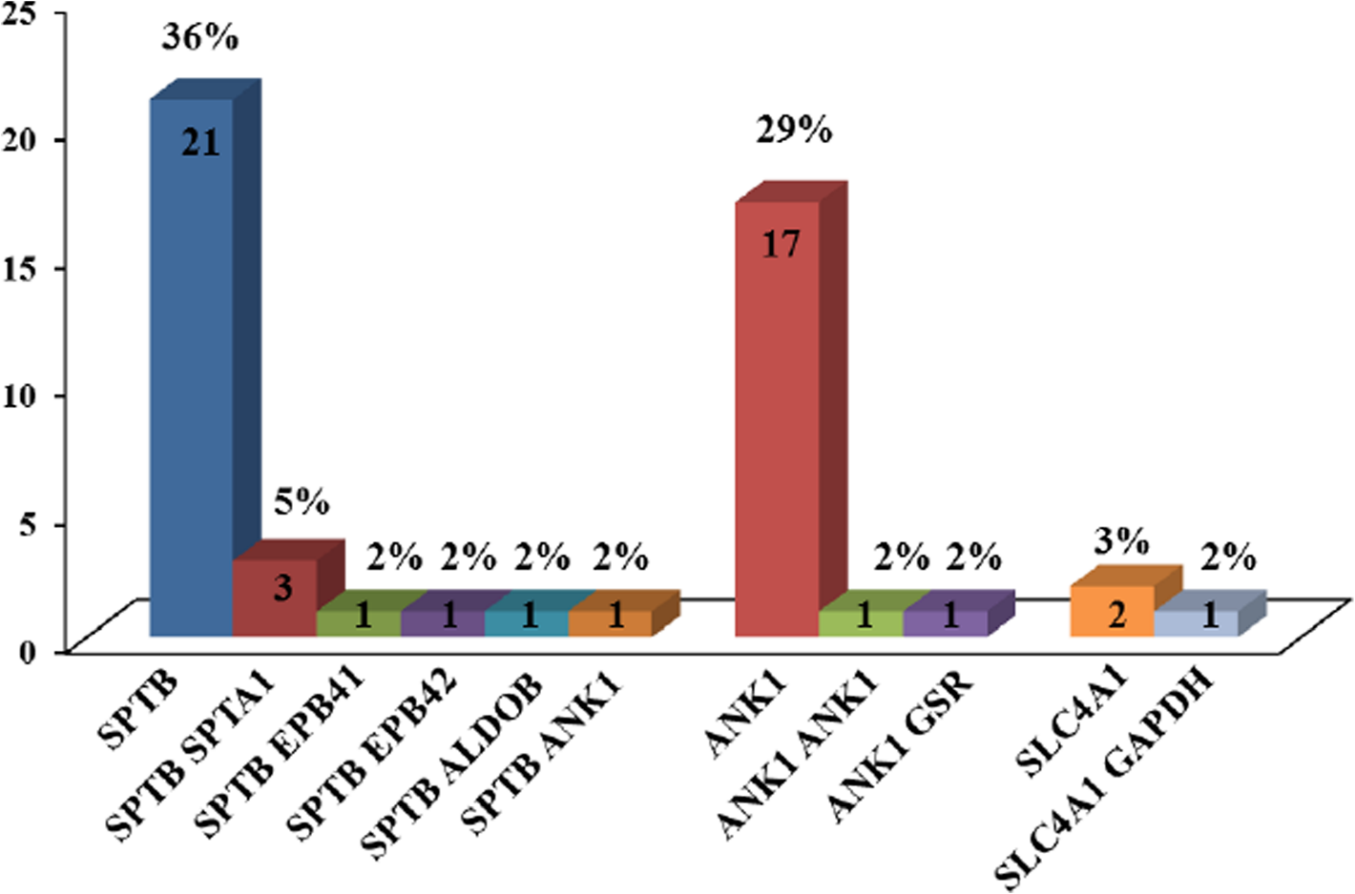
Number of patients with RBC membrane protein-encoding gene mutations. Abbreviations: *SPTB,* spectrin, beta; *SPTA1,* spectrin, alpha 1; *EPB41,* erythrocyte membrane protein band 4.1; *EPB42,* erythrocyte membrane protein band 4.2; *ALDOB*, aldolase B; *ANK1,* ankyrin 1; *GSR*, glutathione reductase; *SLC4A1,* solute carrier family 4, member 1; *GAPDH*, glyceraldehyde-3-phosphate dehydrogenase

Among 59 patients with HS, 50 (84.7%) had at least one mutation in a RBC membrane protein-encoding gene (Fig. 3). Twenty eight patients carried mutations in the *SPTB* gene, and 20 patients had mutations in the *ANK1* gene. Forty patients (67.8%) carried a single mutation, and 10 patients (16.9%) carried two mutations. Among 40 patients with a single mutation, the most frequently mutated genes were *SPTB* and *ANK1*, which were mutated in 21 and 17 patients, respectively. The *SCL4A1* mutation was found in two patients. Among the 10 patients harboring two mutations, one carried two mutations in a single gene (*ANK1*), and three patients carried mutations in both *SPTB* and *SPTA1*. Combinations of mutations in *SPTB* and *ANK1*, *SPTB* and *EPB41,* and *SPTB* and *EPB42* were detected in one patient each. In addition, combination with RBC enzyme-encoding gene mutations were found in three patients [*SLC4A1* and *GAPDH* (glyceraldehyde-3-phosphate dehydrogenase), *ANK1* and *GSR* (glutathione reductase), *SPTB* and *ALDOB* (aldolase B)] (Additional file 1: Table S5).

Nine patients carried no mutation on the RBC membrane protein- or enzyme-encoding genes. Coexisting mutations of *UGT1A1* (UDP glycosyltransferase 1 family, polypeptide A1) gene were detected in 24 of 59 HS patients (40.7%), with *UGT1A1* mutations combined with other gene mutations in 20 patients and without other gene mutation in four patients (Table 2, Additional file 1: Table S6). Total bilirubin level or presence of neonatal jaundice did not differ significantly from those without *UGT1A1* mutations.

**Table 2:** Gene mutations, laboratory tests and clinical characteristics

Patient ID	Membrane gene mutation	Other mutation	OFT	PB spherocytes	Splenectomy	Family history of HS	Severity of HS	Additional tests with positive results
1	*SPTB, EPB41*	*UGT1A1*	NA	♦			▲▲▲	SDS-PAGE (Spectrin)
2	*ANK1*		+	♦♦♦		(HA, father)	▲▲	
3	*SPTB*		NA	♦♦		AD	▲▲▲	Flow cytometry^a^
4	*SPTB*	*UGT1A1*	+	♦♦			▲▲	
5			+	♦♦	●	AD	▲▲▲	SDS-PAGE (Spectrin)
6	*ANK1*		–	♦♦	●		▲▲▲	SDS-PAGE (Spectrin)
7	*SPTB*		+	♦			▲▲▲	
8	*SPTB, SPTA1*		+	♦		AD	▲▲▲	
9	*SPTB, SPTA1*		NA	♦		AD	▲▲▲	Flow cytometry^a^
10			+	♦♦	●	(HA, mother)	▲▲▲	
11^c^	*SPTB*	*UGT1A1*	NA	♦♦♦			▲▲▲	
12	*ANK1*		+	♦♦	●		▲▲▲	
13	*SPTB*	*UGT1A1*	+	♦			▲▲	
14			+	♦	●	AD	▲▲▲	
15	*ANK1* ^b^		NA	♦♦		AD	▲▲	SDS-PAGE (Spectrin)
16	*ANK1* ^b^	*UGT1A1*	NA	♦♦♦		AD	▲▲▲	
17		*UGT1A1*	+	♦♦♦			▲▲	
18	*ANK1*		NA	♦♦♦		AD	▲▲▲	
19	*ANK1*	*UGT1A1, UGT1A1*	+	♦♦			▲▲	
20	*SPTB, SPTA1*	*UGT1A1*	–	♦♦		AD	▲▲▲	
21^c^	*SLC4A1* ^b^	*UGT1A1*	NA	–		(HA, sibling)	▲▲	
22		*UGT1A1*	+	♦		AD	▲▲▲	
23	*SPTB*		+	♦♦♦		AD	▲▲▲	
24		*UGT1A1*	+	♦♦		(HA, mother)	▲▲▲	
25^c^	*ANK1* ^b^		NA	♦♦♦			▲▲	
26	*ANK1*		+	♦♦♦	●	AD	▲▲	
27	*ANK1*		+	♦			▲▲▲	
28	*SPTB*		+	♦♦	●	AD	▲▲	
29	*ANK1* ^b^	*GSR*	+	♦♦			▲▲	
30	*SPTB*	*ALDOB*	+	♦♦♦			▲▲	
31^c^	*SPTB*	–	NA	♦♦♦			▲▲▲	
32	*SLC4A1* ^b^	*UGT1A1, UGT1A1*	+	♦♦♦			▲▲	
33	*SPTB*	*UGT1A1*	+	♦♦	●		▲▲▲	
34	*SPTB*	–	–	♦		AD	▲▲	
35	*SPTB* ^b^ *, EPB42*	*UGT1A1*	+	–			▲▲	Autohemolysis
36	*SPTB*		+	♦♦	●		▲▲	
37	*ANK1*		+	♦♦	●		▲▲	
38		*UGT1A1*	+	♦			▲	SDS-PAGE (Spectrin)
39^c^	*ANK1*	*UGT1A1*	–	♦			▲	
40	*SPTB*		+	♦♦	●		▲▲▲	
41	*ANK1*		+	♦♦♦			▲	
42	*ANK1*		+	♦♦			▲▲▲	
43^c^	*ANK1, ANK1*	*UGT1A1*	NA	♦			▲▲	
44	*SPTB,ANK1*	*UGT1A1*	+	♦			▲▲	
45	*ANK1*	*UGT1A1*	+	♦♦♦			▲▲	
46	*SPTB*	*UGT1A1*	+	♦♦♦			▲▲	
47	*SPTB*		+	♦		(HA, sibling)	▲▲▲	
48^c^	*SPTB* ^b^		NA	♦			▲▲▲	
49^c^	*SPTB*	*UGT1A1*	+	–			▲▲	
50	*ANK1*		+	–		AD	▲▲	
51	*SPTB*	*UGT1A1*	+	♦		AD	▲▲	
52			+	♦♦			▲▲	
53	*ANK1*		+	♦			▲	
54			–	–	●	AD	▲▲▲	
55	*SPTB*		–	♦♦♦		AD	▲	
56	*SLC4A1*	*UGT1A1, GAPDH*	+	♦			▲	
57	*SPTB*		+	♦♦♦		AD	▲▲▲	
58	*SPTB*	*UGT1A1*	+	♦♦		AD	▲▲	
59	*SPTB*		+	♦			▲▲▲	

^a^Flow cytometry (OFT and EMA binding test), ^b^Previously reported variants (see Additional file 1: Table S3), ^c^Eight patients who did not meet the diagnostic criteria of HS without genetic testing

PB spherocytes [20] ♦, 1+; ♦♦, 2+; ♦♦♦, 3+, Severity of HS [8] ▲, mild; ▲▲, moderate; ▲▲▲, severe

Abbreviations: *AD* autosomal dominant, *ALDOB* aldolase B, *ANK1* ankyrin 1, *EPB41* erythrocyte membrane protein band 4.1, *EPB42* erythrocyte membrane protein band 4.2, *GAPDH* glyceraldehyde-3-phosphate dehydrogenase, *GSR* glutathione reductase, *HA* hemolytic anemia, *SLC4A1* solute carrier family 4, member 1, *SPTA1* spectrin, alpha 1, *SPTB* spectrin, beta, *UGT1A1*, UDP glycosyltransferase 1 family, polypeptide A1, *OFT* osmotic fragility test, *NA* not assessable

### Genotype and phenotype correlations in patients with HS

Comparisons of laboratory findings and clinical characteristics showed no significant differences in hematologic parameters, hemolysis markers, iron status parameters, sex, family history of HS, number of splenectomized patients, and disease severity according to the gene mutation type and number of mutation or presence of *UGT1A1* mutation (Table 1, Additional file 1: Table S6).

Among 59 patients with HS, nine patients (15.3%) without mutation associated with RBC membrane protein-encoding genes showed similar baseline characteristics in most aspects as compared with those with mutations (Table 1). Median age of patients without mutation was 8 years, and the proportion of family history, clinical symptoms, grading of peripheral spherocytes, and OFT results did not differ significantly from those with mutation.

### Intercorrelations between gene mutations and laboratory findings: OFT, the presence of spherocytes in PBS, and gene mutations

**Fig. 4:**
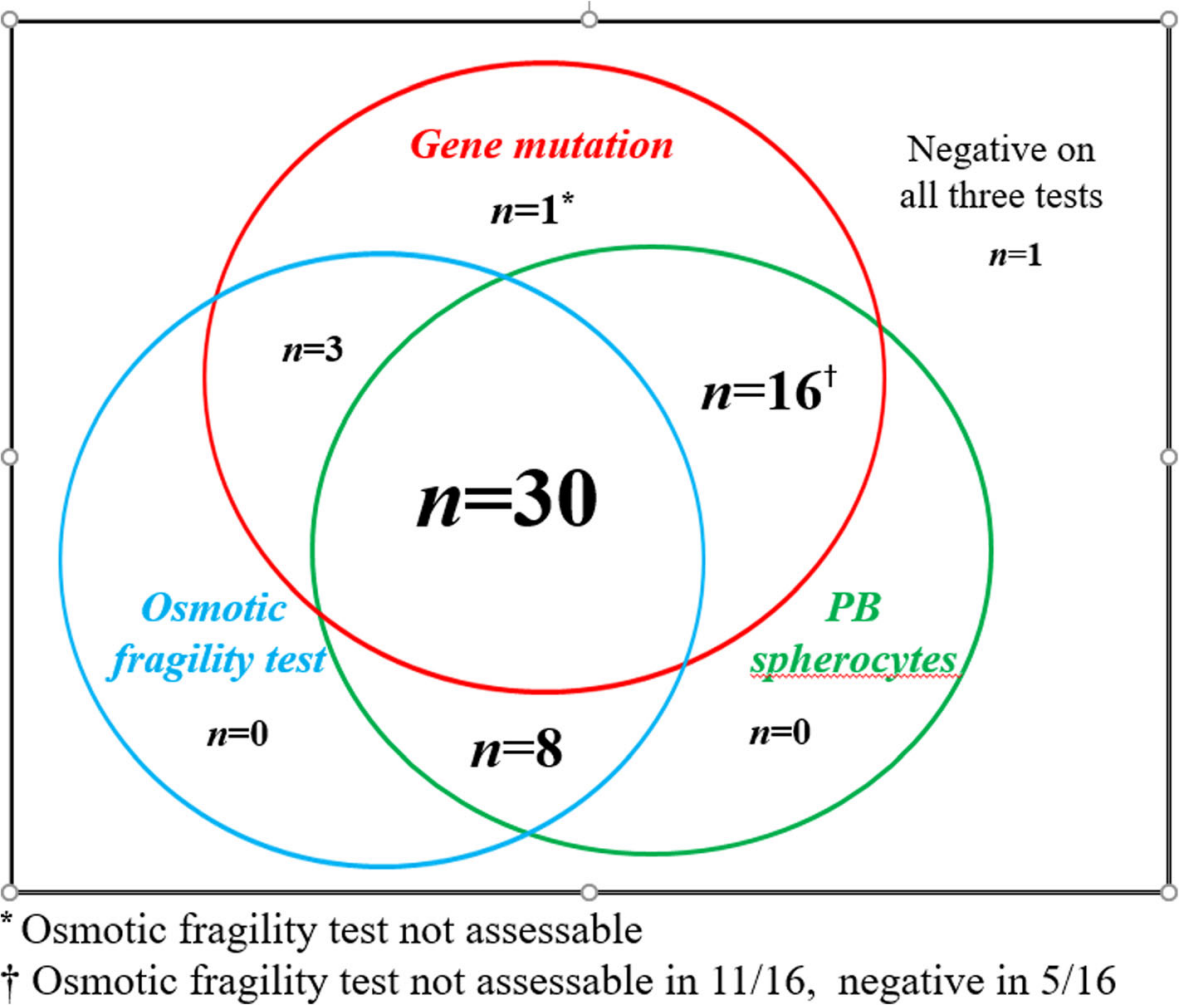
A diagram showing the number of patients with positive results of gene mutation, osmotic fragility test, and peripheral blood (PB) spherocytes in 58 of 59 patients with HS. One of 59 patients who had anemia and family history of HS showed negative result on all three tests

The results of genetic test were matched with routine diagnostic tests for HS including OFT and the presence of spherocytes in PBS (Table 3, Fig. 4). Among 59 patients with clinical HS, results of NaCl induced OFT (room temperature and/or 24 h incubated) was available in 47 patients and 41 of them (87.2%) showed positive results (Additional file 1: Figure S2). Thirty three of 47 patients (70.2%) showed positivity in both OFT and gene test, while one patients (2.1%) showed negative results in both OFT and gene test. In six out of 47 patients (12.7%) with negative OFT, five carried mutations in RBC membrane protein-encoding genes. Among 38 patients harboring HS-related gene mutations, 33 showed positive OFT (86.8%).

**Table 3:** Comparison of OFT, PBS and gene test results in patients with HS

		RBC membrane protein-encoding genes	
		No. of patients with mutation (%)	No. of patients without mutation (%)
OFT (*n* = 47)	Positive	33 (70.2)	8 (17.0)
	Negative	5 (10.6)	1 (2.1)
PBS (*n* = 59)	Positive	46 (78.0)	8 (13.6)
	Negative	4 (6.8)	1 (1.7)

Abbreviation: *OFT* osmotic fragility test, *PBS* peripheral blood cell smear

Spherocytes in PBS were present in 54 of 59 patients (91.5%). Among five patients without spherocytes in PBS, four carried mutations in RBC membrane protein-encoding genes (Additional file 1: Table S7). One of 59 patients who had anemia and family history of HS showed negative results on all three tests.

## Discussion

Using multi-gene target sequencing, 50 of 59 patients (84.7%) of clinically diagnosed HS proved to be molecular HS and three patients harbored coexisting gene mutations of RBC enzymes (*ALDOB, GAPDH,* and *GSR*) in this study. Mutations of six kinds of RBC membrane protein-encoding genes (total 54 variants) were detected in order of *SPTB*, *ANK1*, *SLC4A1*, *SPTA1*, *EPB41*, and *EPB42*.

To find whether there is an ethnic difference in HS related variants, we reviewed the literatures on the reports of HS related mutations in comparison with the results of the present study, although the methods are different among reported mutations of HS. Table 4 shows summary of comparison among previous reports by NGS [22–24]. With regards to the frequency of mutated gene, the *SPTA1* mutation was the most common followed by the *SPTB* mutation in the reports from the United States [22, 23]. Meanwhile, a study in Netherland revealed that the *ANK1* mutation was the most common mutation followed by the *SPTA1* mutation [24]. In the present study, *SPTB* mutations was the most common mutation, followed by *ANK1* mutations. Particularly noteworthy, *SPTA1* mutations was rarely detected, compared to that of the United States. Briefly, mutation frequency by NGS study in Korean was different from those of Caucasian. Korean patients with HS showed higher frequency of *ANK1* mutation. Consistent with our study, another study in Korea reported that 25 patients with HS carried one heterozygous mutation of *ANK1* (*n* = 13) or *SPTB* (*n* = 12) but none carried mutations in *SPTA1*, *SLC4A1*, or *EPB42* by Sanger sequencing [25]. Previous molecular testing demonstrated that mutations in the *ANK1, SPTB, SLC4A1, SPTA1*, and *EPB42* genes account for 60, 10, 15, 10, and 5% cases of HS, respectively, in the United States and Europe [26, 27].

**Table 4:** NGS results of RBC membrane protein-encoding genes in patients with HS

RBC membrane-encoding gene	USA 1 [22]	USA 2 [23]	Netherlands [24]	Korea (this study)
No. of patients with mutation (%)	10/20^a^ (50.0)	16 /19^b^ (84.2)	52 /66 (78.9)	50/59 (84.7)
No. of total mutations	13	21	73	57
No. of different variants	11	15	53	54
*ANK1*	*1*	*3*	*14*	*19*
*SPTA*	*6*	*5*	*25*	*2*
*SPTB*	*4*	*4*	*8*	*28*
*SCL4A1*	*0*	*3*	*4*	*3*
*EBP41*	*NA*	*0*	*1*	*1*
*EBP42*	*NA*	*0*	*1*	*1*

^a^including 2 patients suspected having hereditary elliptocytosis

^b^including patients with diagnosed as HHA

Abbreviation; *NA* not assessable

Ethnic differences in RBC membrane protein defects were also reported in previous studies according to sodium dodecyl sulfate polyacrylamide gel electrophoresis (SDS-PAGE) analyses (Table 5) [9, 16, 28–32]. A Korean study in 2000 [28] reported that protein 4.2 defects were detected at a higher frequency than those of band 3 in the United States and Europe. That study also reported that most defects were found in ankyrin 1 according to SDS-PAGE analysis, whereas most mutations were detected in the *SPTB* followed by *ANK1*, according to our NGS results. Additionally, protein defects were not observed was nine out of 27 patients (33.3%) [28]. Meanwhile, single defects in band 3 and spectrin constitute the primary variants reported in Italy [9, 16], and a combined defect in spectrin/ankyrin is frequently detected in patients in the United States and Spain [6, 29, 30]. Regarding to the incidence of HS, an incidence of Japan is highest among Asian countries, and the defect in the 4.2 protein in Japan is more frequent as compared to the United States and Europe [31, 32]. Those different profiles of HS among countries might be due to complexity associated with SDS-PAGE methods and lack of objectiveness in the interpretation of the results. The interpretation of SDS-PAGE is based on the comparison with normal healthy control. For that reason, the standardization is not possible and the comparison of SDS-PAGE results cannot give a meaningful conclusion. By contrast, nucleotide sequence analysis gives us straightforward results, and the interpretation of results is objective.

**Table 5:** Literature review on SDS-PAGE results of RBC membrane protein abnormalities in patients with HS (%)

RBC membrane protein	Italy2[16] (*n* = 87)	Italy1[9] (*n* = 300)	USA2[6] (*n* = 55)	USA1[29] (*n* = 166)	Spain[30] (*n* = 62)	Japan2*[31] (*n* = 60)	Japan1[32] (*n* = 47)	Korea[28] (*n* = 27)
Band 3	23 (26)	158 (53)	10 (18)	38 (23)	0	(20)	15 (32)	3 (11)
Spectrin only	36 (41)	98 (33)	7 (13)	0	19 (31)	0	8 (15)	2 (7)
Ankyrin only	0	13 (4)^†^	0	0	4(6)	(7)	1 (2)	8 (30)
Spectrin/ankyrin	16 (18)		6 (11)	100 (60)	34 (55)	0	1 (2)	1 (4)
Other combination	–	–	–	–	–	–	15 (34)	–
4.2 protein	6 (7)	2 (1)	0	3 (2)	0	(45)	3 (6)	4 (15)
Undetected	6 (7)	29 (10)	32 (58)	25 (15)	5 (8)	(28)	4 (9)	9 (33)

*Only % without the number of the patients was presented in this study

^†^Including both Ankyrin only and Spectrin/ankyrin

Inherited pattern of HS differs depending on the gene. In most HS patients, inheritance is AD and each of HS patients has a unique mutation [11]. However, *SPTA1* or *EPB42* mutation is inherited with AR pattern. Rarely, double dominant HS due to defects in *SLC4A1* or *SPTB* are reported [33], which results in fetal death or severe transfusion-dependent hemolytic anemia presenting in the neonatal period. *SPTB* and *SPTA1* mutations can be AD or de novo, whereas *ANK1*mutation can be AD, AR, or de novo. *SLC4A1* mutation is AD and *EPB42* is AR. Inherited pattern is not clearly revealed in *EPB41*. Of note, all the significant variants in RBC membrane protein-encoding genes are heterozygous. Hence, mutations of genes inherited in AR pattern such as *EPB41* and *EPB42* gene possibly cannot be a direct cause of HS, requiring additional mutation to cause hemolytic phenotype. In the present study, two patients harboring *EPB41* and *EPB42* mutations also carried another mutation in the *SPTB* gene (*EPB41* and *SPTB*, *EPB42* and *SPTB* in each patient).

Interestingly, concurrent mutations of genes encoding RBC enzymes (*ALDOB, GAPDH*, and *GSR*) were detected along with heterozygous mutations of RBC membrane protein-encoding genes in three patients. Further analysis of enzyme activities in these patients is necessary for validation. Of the 59 patients with HS examined in this study, 24 (40.7%) had significant *UGT1A1* variants. It was reported that a polymorphism of *UGT1A1* gene promoter homozygous insertion of TA pairs (genotype UGT1A1*28/*28) might results in a decrease in bilirubin glucuronidation activity, leading to hyperbilirubinemia and late complication of patients with HS, such as development gallstones [34, 35]. In contrast, there are debates on the late impact of genotype of *UGT1A1* [36]. However, a polymorphism of *UGT1A1* gene promoter was not included in this study. Based on the results of the present study showing high frequency of *UGT1A1* variant with low enzymatic activity, we infer that genotyping of *UGT1A1* polymorphism might help to predict the development of gallstones in HS.

The laboratory diagnosis of HS routinely relies on the presence of spherocytes in PBS, OFT, and more recently EMA binding test [10, 11, 37, 38]. Yet, there is no single test that can confirm HS. We have matched the results of genetic test with those of routine diagnostic tests (Table 3). Among 50 patients harboring mutations of encoding RBC membrane protein, 86.8% showed positive OFT, while 70.2% of clinical HS showed positive OFT. On the contrary, eight patients (17.0%) with positive OFT result revealed no mutation of membrane genes, and five (10.6%) with negative OFT proved to harbor membrane gene mutation. Regarding to spherocytes, four of 50 patients (8%) harboring membrane gene mutation did not show spherocytes in PBS. We retrospectively reviewed PBS to determine the presence of spherocytes in those four patients who did not show spherocytes in PBS but with RBC membrane protein-encoding gene mutations. However, we could not detect additional spherocytes. Conclusively, OFT and spherocytes in PBS can be used in conjunction with genetic test for the -diagnosis of HS, giving higher sensitivity and specificity.

With regards to the genotype-phenotype relationship, we could not find any correlation between the genetic test results and clinical characteristics including disease severity, mean hemoglobin concentrations, splenomegaly, gallstones, aplastic crisis and bilirubin levels according to mutations of four genes (*SPTB, ANK1, SPTA1,* and *SLC4A1*), except *EPB41* and *EPB42*, which were found in only one patient each, However, one study reported that anemia was most severe in HS patients with mutations on the *ANK1* spectrin-binding domain and splenectomy was more frequently performed in patients with *ANK1* mutations than in those with *SPTB* mutations [25]. In addition, the other reported that hemoglobin concentration was slightly lower in patients with spectrin deficiency than with band 3 deficiency [39].

Other NGS study on RBC membrane diseases reported similar results (86.3%, 44 of 51 patients) [24]. This finding suggested a close correlation between clinical diagnosis and gene mutations. In the present study, molecular test could detect additional HS which could be missed without molecular test (Fig. 4). Furthermore, molecular test would be an effective method for neonates or transfused individuals, since the result of OFT and spherocytes in PBS can be unreliable, especially when the patients are transfused [11]. Collectively, our results suggest that mutation analyses will complement with other conventional tests for accurate diagnosis of HS. We consider the molecular test needs to be integrated to the diagnostic criteria of HS.

The limitation of this study is that we did not perform the analysis on RBC membrane protein as a validation. Instead, we simulated 3-D spatial structure of protein encoding mutated genes, predicting the effects of gene mutations in silico. Although exact changes in protein structure cannot be predicted based on 3-D spatial structure, large-scale modification of the protein due to frame shift or nonsense mutations can be visualized and subsequent functional changes can be expected from structure analysis. Further family study or functional studies using knockout mice needs to be conducted to validate the significance of variants. Another limitation is that we could not match the results of EMA binding test with genetic results, since our study was done retrospectively. Nine patients who did not harbor gene mutation of RBC membrane protein (Additional file 1: Table S8), satisfied the diagnostic criteria of HS suggested in the guideline [11]. Though they satisfied those criteria, there are two possibilities that they have other forms of hemolytic anemia or other membrane gene mutations that is not included in our multi-gene panel (e.g. channel defects such as *KCNN4* as found in hereditary stomatocytosis) [40].

When we target the most frequent mutations only, composition of gene panel with genes over 10% frequency (*SPTB* and *ANK1*) will cover 94% (47 of 50 patients) of the diagnosis of HS. This could provide a cheaper and more convenient method than current strategies for diagnosis of HS. Regarding to the diagnostic guidelines suggested by international working parties, we suggest that genetic test should be conducted at least in patients without clues of laboratory tests in spite of clinically suspected HS.

## Conclusions

This constitutes the first large-scaled genetic study of Korean patients with HS. We detected 54 significant HS-related mutations, including 46 novel mutations in RBC membrane protein-encoding genes. We demonstrated that multi-gene target sequencing is sensitive and feasible that can be used as a powerful tool for diagnosing HS. Considering the discrepancies between clinical and molecular diagnoses, use of molecular genetics analysis provides an effective method for improving the accuracy of HS diagnosis.

## Additional file

Additional file 1: **Figure S1.** Significant variants diagrams for *UGT1A1* gene. **Figure S2.** Results of NaCl induced OFT. **Table S1.** Multi-gene panel for targeted sequencing. **Table S2.** List of protein simulation templates. **Table S3.** List of significant variants detected in RBC membrane protein-encoding genes. **Table S4.** Primer sets for all significant variants in RBC membrane protein-encoding genes. **Table S5.** List of significant variants detected in RBC enzyme-encoding genes among patients with HS. **Table S6.** List of *UGT1A1* gene variants in patients with HS in Korea. **Table S7.** Clinical characteristics of patients with HS without peripheral blood spherocytes. **Table S8.** Patients without RBC membrane-encoding gene mutation. (DOCX 114 kb)

## Abbreviations

AD: Autosomal dominant
ALDOB: Aldolase B
ANK1: Ankyrin 1
AR: Autosomal recessive
CDA: Congenital dyserythropoietic anemia
EMA: Eosin-5-maleimide
EPB42: Erythrocyte membrane protein band 4.2
GAPDH: Glyceraldehyde-3-phosphate dehydrogenase
GSR: Glutathione reductase
HHA: Hereditary hemolytic anemia
HS: Hereditary spherocytosis
ICSH: International Council for Standardization in Haematology
IRB: Institutional Review Board
KHHAWP: The Korean Hereditary Hemolytic Anemia Working Party
LDH: Lactate dehydrogenase
NA: Not assessable
NGS: Next-generation sequencing
OFT: Osmotic fragility test
PBS: Peripheral blood smear
PNH: Paroxysmal nocturnal hemoglobinuria
SLC4A1: Solute carrier family 4, member 1
SNP: Single nucleotide polymorphism
SOP: Standard operating procedure
SPTA1: Spectrin, alpha 1
SPTB: Spectrin, beta
TIBC: Total iron-binding capacity
