# Supplementary material for: Molecular diagnosis of hereditary spherocytosis by multi-gene target sequencing in Korea: matching with osmotic fragility test and presence of spherocyte
Source: Orphanet J Rare Dis. 2019 May 23;14:114. doi: 10.1186/s13023-019-1070-0 (PMC6533652; doi:10.1186/s13023-019-1070-0)
Supplement: Supplementary file 1 — Figure S1. Significant variants diagrams for UGT1A1 gene. Figure S2. Results of NaCl induced OFT. Table S1. Multi-gene panel for targeted sequencing. Table S2. List of protein simulation templates. Table S3. List of significant variants detected in RBC membrane protein-encoding genes. Table S4. Primer sets for all significant variants in RBC membrane protein-encoding genes. Table S5. List of significant variants detected in RBC enzyme-encoding genes among patients with HS. Table S6. List of UGT1A1 gene variants in patients with HS in Korea. Table S7. Clinical characteristics of patients with HS without peripheral blood spherocytes. Table S8. Patients without RBC membrane-encoding gene mutation. (DOCX 114 kb) [file 13023_2019_1070_MOESM1_ESM.docx]

**Additional file 1**

**Figure S1** Significant variants diagrams for *UGT1A1* gene

**Figure S2** Results of NaCl induced OFT

**Table S1** Multi-gene panel for targeted sequencing

**Table S2** List of protein simulation templates

**Table S3** List of significant variants detected in RBC membrane protein-encoding genes

**Table S4** Primer sets for all significant variants in RBC membrane protein-encoding genes

**Table S5** List of significant variants detected in RBC enzyme-encoding genes among patients with HS

**Table S6** List of *UGT1A1* gene variants in patients with HS in Korea

**Table S7** Clinical characteristics of patients with HS without peripheral blood spherocytes

**Table S8** Patients without RBC membrane-encoding gene mutation

**
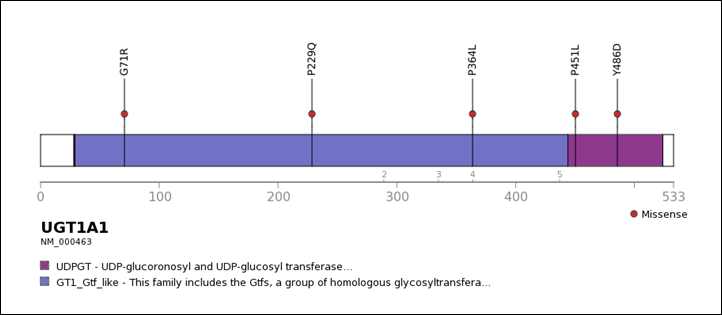
**

**Figure S1** Significant variants diagrams for *UGT1A1* gene

***n*=17**

**(+) in 17 patients**

Both in RT & 24 hour

Patients ID

8, 12, 14, 22, 29, 30,

40, 41, 42, 44, 52, 56

Only in 24 hour

Patients ID

10, 27, 38, 47, 58

***n*=4**

**(+) in 4 patients**

Patients ID

19, 33, 37, 59

***Room temperature OFT***

***24 hour incubated OFT***

***n*=26**

**(+) in 20 patients**

Patients ID

2, 4, 5, 6, 7, 14, 17, 23, 24, 26, 28,

32, 35, 36, 45, 46, 49, 50, 51, 53

**(-) in 6 patients**

Patients ID

20, 34, 39, 54, 55, 57

***OFT not assessed n*=12**

Patients ID 1, 3*, 9*, 11, 15, 16, 18, 21, 25, 31, 43, 48

* Flow cytometric OFT and EMA binding test were done in two patients.

**Figure S2** Results of NaCl induced OFT

* Abbreviations: OFT, osmotic fragility test; RT, room temperature**Table S1** Multi-gene panel for targeted sequencing

|  | **Protein** | **Gene** | **Location** |
| --- | --- | --- | --- |
| **RBC membrane protein** | Spectrin alpha, erythrocytic 1 | *SPTA1* | 1q21 |
|  | Spectrin beta, erythrocytic | *SPTB* | 14q23-q24.2 |
|  | Ankyrin 1 | *ANK1* | 8p11.1 |
|  | Band 3 | *SLC4A1* | 17q21.31 |
|  | Protein 4.1 | *EPB41* | 1p33-p32 |
|  | Protein 4.2 | *EPB42* | 15q15-q21 |
|  | p55 | *MPP1* | Xq28 |
|  | Beta-adducin | *ADD2* | 2p13.3 |
|  | Glycophorin A | *GYPA* | 4q31.21 |
|  | Glycophorin B | *GYPB* | 4q31.21 |
|  | Glycophrins C/D | *GYPC* | 2q14-q21 |
|  | Stomatin | *STOM* | 9q34.1 |
|  | Aquaporin | *AQP1* | 7p14 |
|  | Dematin | *DMTN* | 8p21.1 |
|  | Beta-actin | *ACTB* | 7p22 |
|  | Tropomodulin 1 | *TMOD1* | 9p22.3 |
|  | Tropomyosin 3 | *TPM3* | 1q21.2 |
| **RBC enzyme** | Hexokinase | *HK1* | 10q22 |
|  | Glucose phosphate isomerase | *GPI* | 19q13.1 |
|  | Enolase | *ENO1* | 1p36.2 |
|  | Lactate dehydrogenase | *LDHB* | 12p12.2-p12.1 |
|  | Glutathione reductase | *GSR* | 8p21.1 |
|  | Acetylcholinesterase | *ACHE* | 7q22 |
|  | Glyceraldehyde-3-phosphate dehydrogenase | *GAPDH* | 12p13 |
|  | Phosphofructokinase | *PFKM* | 12q13.3 |
|  | Phosphoglycerate kinase | *PGK1* | Xq13.3 |
|  | Pyruvate kinase | *PKRL* | 1q21 |
|  | Glucose-6-phosphate dehydrogenase | *G6PD* | Xq28 |
|  | Adenylate kinase | *AK1* | 9q34.1 |
|  | Adenosine deaminase | *ADA* | 20q13.12 |
|  | Pyrimidine 5'-nucleotidase (CMP) | *CMPK1* | 1p32 |
|  | Pyrimidine 5'-nucleotidase (UMP) | *NT5C3A* | 7p14.3 |
|  | Aldolase A | *ALDOA* | 16p11.2 |
|  | Aldolase B | *ALDOB* | 9q21.3-q22.2 |
|  | Triosephosphate isomerase | *TPI1* | 12p13 |
|  | 6-phosphogluconic dehydrogenase | *PGD* | 1p36.22 |
|  | Glutathione peroxidase | *GPX1* | 3p21.3 |
| **Thalassemia** | Hemoglobin alpha1 | *HBA1* | 16p13.3 |
|  | Hemoglobin alpha2 | *HBA2* | 16p13.3 |
|  | Hemoglobin beta | *HBB* | 11p15.5 |
| **CDAII** | Sec23 homolog B | *SEC23B* | 20p11.23 |
| **Gilbert syndrome** | UDP glucuronosyltransferase 1 family | *UGT1A1* | 2q37 |
| **PNH** | Phosphatidylinositol Glycan Anchor Biosynthesis, Class A | *PIGA* | Xp22.1 |

**Table S2** List of protein simulation templates

| **Protein** | **UnitProt  number** | **Template** | **Start position** | **End position** | **Length** |
| --- | --- | --- | --- | --- | --- |
| Spectrin  beta chain | P11277 | Model 8 from SWISSMODEL | 55 | 600 | 546 |
|  |  | Model 32 from SWISSMODEL | 652 | 951 | 300 |
|  |  | Experimental structure 1S35 | 1064 | 1275 | 212 |
|  |  | Model 25 from SWISSMODEL | 1277 | 1580 | 304 |
|  |  | Experimental structure 3KBU | 1582 | 1906 | 325 |
|  |  | Experimental structure 3LBX | 1901 | 2084 | 184 |
| Ankyrin-1 | P16157 | Model 5 from SWISSMODEL | 9 | 789 | 781 |
|  |  | Experimental structure 1N11 | 405 | 812 | 408 |
|  |  | Experimental structure 3UD1 | 912 | 1233 | 322 |
|  |  | Experimental structure 2YQF | 1394 | 1497 | 104 |
| Band 3 | P02730 | Experimental structure 1BH7 | 803 | 835 | 33 |
| Spectrin  alpha chain | P02549 | Model 4 from SWISSMODEL | 1606 | 1816 | 211 |
| Protein 4.1 | P11171 | Model 1 from SWISSMODEL | 208 | 519 | 312 |
| Protein 4.2 | P16452 | Model 2 from: SWISSMODEL | 5 | 689 | 685 |
| Aldolase B,  fructose-bisphosphate | P05062 | Experimental structure 1QO5 | 2 | 364 | 363 |
| Glucose-6-phosphate  dehydrogenase | P11413 | Experimental structure 1QKI | 2 | 515 | 514 |
| Glyceraldehyde-3- phosphate dehydrogenase | P04406 | Experimental structure 4WNC | 1 | 335 | 335 |
| Glutathione reductase | P00390 | Experimental structure 3DJG | 45 | 522 | 478 |

**Table S3** List of significant variants detected in RBC membrane protein-encoding genes

| **Gene** | **Chromosome** | **Position** | **SNP ID** | **Variant (nucleotide)** | **Variant (amino acid)** | **Zygosity** | **ACMG Classification** | **Patient ID** |
| --- | --- | --- | --- | --- | --- | --- | --- | --- |
| *SPTB* | Chr14 | 65289812 | rs121918651 | c.1A>G^†^ | p.Met1Val | Heterozygous | Pathogenic | 35 |
|  |  | 65271770 | . | c.187T>C | p.Trp63Arg | Heterozygous | Uncertain significance | 9 |
|  |  | 65271749 | . | c.208C>T | p.Arg70* | Heterozygous | Likely pathogenic | 33 |
|  |  | 65270438 | . | c.360_361insTC |  | Heterozygous | Likely pathogenic | 44 |
|  |  | 65270423 | . | c.376C>T | p.Gln126* | Heterozygous | Likely pathogenic | 40 |
|  |  | 65270327 | . | c.472C>T | p.Gln158* | Heterozygous | Likely pathogenic | 56 |
|  |  | 65268479 | . | c.639_640insG |  | Heterozygous | Likely pathogenic | 8 |
|  |  | 65268475 | . | c.643_644insC |  | Heterozygous | Likely pathogenic | 13 |
|  |  | 65268471 | . | c.647+1G>C |  | Heterozygous | Uncertain significance | 1 |
|  |  | 65267587 | . | c.764-1G>T |  | Heterozygous | Uncertain significance | 48 |
|  |  | 65260366 | . | c.2014delG |  | Heterozygous | Likely pathogenic | 50 |
|  |  | 65259848 | . | c.2533C>T^‡^ | p.Gln845* | Heterozygous | Pathogenic | 48 |
|  |  | 65253442 | . | c.3241C>T | p.Gln1081* | Heterozygous | Likely pathogenic | 31 |
|  |  | 65253216 | . | c.3466_3467insA |  | Heterozygous | Likely pathogenic | 30 |
|  |  | 65253192 | . | c.3489_3490delCC |  | Heterozygous | Likely pathogenic | 28 |
|  |  | 65246649 | . | c.4267C>T | p.Arg1423* | Heterozygous | Likely pathogenic | 46 |
|  |  | 65246625 | . | c.4291C>T | p.Arg1431* | Heterozygous | Likely pathogenic | 55 |
|  |  | 65246594 | . | c.4321delC |  | Heterozygous | Likely pathogenic | 47 |
|  |  | 65245922 | . | c.4515delC |  | Heterozygous | Likely pathogenic | 3 |
|  |  | 65242097 | . | c.4577_4586del |  | Heterozygous | Pathogenic | 23 |
|  |  | 65241843 | . | c.4842G>C | p.Lys1614Asn | Heterozygous | Uncertain significance | 4 |
|  |  | 65241215 | . | c.4873C>T | p.Arg1625* | Heterozygous | Likely pathogenic | 59 |
|  |  | 65240129 | . | c.4987A>T | p.Arg1663* | Heterozygous | Likely pathogenic | 34 |
|  |  | 65240100 | . | c.5015delA |  | Heterozygous | Likely pathogenic | 7 |
|  |  | 65239958 | . | c.5153_5154insG |  | Heterozygous | Likely pathogenic | 11 |
|  |  | 65237602 | . | c.5798+1G>A |  | Heterozygous | Uncertain significance | 20 |
|  |  | 65235838 | . | c.5938-2A>G |  | Heterozygous | Uncertain significance | 36 |
|  |  | 65235751 | . | c.6022+1G>A |  | Heterozygous | Uncertain significance | 57 |
| *ANK1* | Chr8 | 41583380 | . | c.511A>T | p.Lys171* | Heterozygous | Likely pathogenic | 12 |
|  |  | 41583298 | . | c.593A>G | p.Asn198Ser | Heterozygous | Uncertain significance | 44 |
|  |  | 41580722 | . | c.830A>G^†^ | p.His277Arg | Heterozygous | Pathogenic | 15 |
|  |  | 41580696 | . | c.856C>T | p.Arg286* | Heterozygous | Likely pathogenic | 41 |
|  |  | 41566369 | . | c.1924delG |  | Heterozygous | Likely pathogenic | 45 |
|  |  | 41563729 | . | c.2029C>T | p.Gln677* | Heterozygous | Likely pathogenic | 6 |
|  |  | 41563662 | . | c.2095_2096insT |  | Heterozygous | Likely pathogenic | 49 |
|  |  | 41561982 | . | c.2106C>A | p.Tyr702* | Heterozygous | Likely pathogenic | 43 |
|  |  | 41561627 | . | c.2227C>T | p.Gln743* | Heterozygous | Pathogenic | 19,26 |
|  |  | 41559135 | . | c.2390_2393delTAGT^†^ |  | Heterozygous | Pathogenic | 25 |
|  |  | 41554101 | . | c.2739delT |  | Heterozygous | Likely pathogenic | 27 |
|  |  | 41554038 | . | c.2803C>T | p.Arg935* | Heterozygous | Likely pathogenic | 42 |
|  |  | 41552280 | . | c.3157C>T^†^ | p.Arg1053* | Heterozygous | Pathogenic | 29 |
|  |  | 41552202 | . | c.3235G>T | p.Glu1079* | Heterozygous | Likely pathogenic | 37 |
|  |  | 41550270 | . | c.3754C>T^†^ | p.Arg1252* | Heterozygous | Pathogenic | 16 |
|  |  | 41547753 | . | c.4095delC |  | Heterozygous | Likely pathogenic | 2 |
|  |  | 41547746 | . | c.4103A>T | p.Lys1368Met | Heterozygous | Uncertain significance | 43 |
|  |  | 41543669 | . | c.4387_4390delAACA |  | Heterozygous | Pathogenic | 18,52 |
|  |  | 41529900 | . | c.5066_5067delTG |  | Heterozygous | Likely pathogenic | 39 |
| *SLC4A1* | Chr17 | 42334818 | . | c.1526G>T | p.Gly509Val | Heterozygous | Uncertain significance | 55 |
|  |  | 42330519 | rs373916826 | c.2278C>T^†^ | p.Arg760Trp | Heterozygous | Pathogenic | 32 |
|  |  | 42328845 | . | c.2423G>A^†^ | p.Arg808His | Heterozygous | Pathogenic | 21 |
| *SPTA1* | Chr1 | 158636139 | . | c.2187C>G | p.His729Gln | Heterozygous | Uncertain significance | 20 |
|  |  | 158607919 | rs182430449 | c.5093T>C | p.Val1698Ala | Heterozygous | Pathogenic | 8,9 |
| *EPB41* | Chr1 | 29342222 | rs189183599 | c.848C>T | p.Thr283Ile | Heterozygous | Uncertain significance | 1 |
| *EPB42* | Chr15 | 43501559 | rs368756068 | c.835G>A | p.Val279Met | Heterozygous | Uncertain significance | 35 |

**^†^** previously reported variants**Table S4** Primer sets for all significant variants in RBC membrane protein-encoding genes

| **Gene** | **Forward** | **Reverse** | **Tm ( ^o^C)** | **Product size** |
| --- | --- | --- | --- | --- |
| *SPTB* | AACAAACGACCCAGAGACCC | TGTGAGAATGCCCTCCAACC | 60 | 436 |
|  | CCTCTGTTCTGGTGGCATGT | GAGAGACCAGTCAGCCACAC | 60 | 517 |
|  | GGAGGGTGGGTGCTTATGAC | GGGGGAGGAGGCTAAAAAGC | 60.2 | 570 |
|  | GCCTCTGTGTGTGGTTAGCT | TTGTACAGAGGCAGGGAGGT | 60.1 | 511 |
|  | ACACTCTTCCATTCTTGCTCTGT | GACATGGGTCTCAGGTGGAC | 59.8 | 507 |
|  | TGAGGAGCTGAGCAACATGG | CTTGTGCTTTTTCCCCAGGG | 59.6 | 502 |
|  | GGCTGCTCTCTGGTGAAGAT | AACAAAGGACATCCCAGGGC | 59.9 | 502 |
|  | ACCCTGAGCAGAAGGAGGAT | GCCCTGAATGCCTCTCTACC | 60 | 507 |
|  | ACCCCTTTTGCAGTGGGTAG | TAAGGGGTGAGGTGACCAGT | 59.9 | 632 |
|  | CCCACTGTCTGACTAGGAGC | TGGAGACCCCAAAGCTACCA | 59.5 | 452 |
|  | CCTGAGGGTGCAAGAATGGT | CCAGGTAGTACTGCTGTGCC | 60 | 438 |
|  | GATCGACTGCCAGGACCTTG | AGTGGGTAAACAGGAGGCAC | 60 | 434 |
|  | TCCAGAGAGGAAGGTAGGGC | ATCTGAAGGGACGTTGGCTG | 60 | 448 |
|  | GCATATGTTGGCTCCCTGGA | GGGGTTACAGGTGTCACCAG | 60 | 618 |
|  | GCAGAGGCCATCCAGAACAA | CCTGACTCTGGAGCGATTGG | 60.2 | 448 |
|  | GTGCACACACTTTGGAGCTG | GCACGCTGGCTAGTTAGAGT | 60 | 578 |
| *ANK1* | TTTGGTGGAGGTAGGTCCCT | AAGAGCACGTTTCTGGGCAT | 60 | 518 |
|  | GTGCAGTGGGAGGCAATAGT | GTGGCTGCTTTTCTGCTACG | 60 | 561 |
|  | CAAGCCTGAGGAGCTGTCTC | TGAAATGTACGCAGGGCTGT | 60 | 533 |
|  | CAGGAAGAGCTTTGCCTGGA | GCTTTTGTGTGGATCTGGGG | 59.6 | 554 |
|  | TCATGTGATGCCCAGCTCAG | GGCCCTAGACACGTGCATTA | 60 | 589 |
|  | CAGCAGTGGAGTGGATGGG | AGTCAATGTTCCCTCACCTAAGT | 59.6 | 502 |
|  | ACGTGGCACCTGGTTTCTAG | GCAAGCTCACCTCAGGAACT | 60 | 532 |
|  | TGTGTGAGAGGATGCTCAGC | GCACCAGACAAAAGTGTGGG | 59.7 | 536 |
|  | CTCCCGTCTGGATGGAAAGG | CCTCGTCTACAGAGGGAGGA | 59.7 | 404 |
|  | TTCCCTAAACCAGACAGCCG | CCAGCACTGCACTGCAAATT | 59.9 | 412 |
|  | CTCAGAGCTCCCTGTGCTC | GTGGGGACTACAGGCATGAG | 59.7 | 530 |
|  | TTGTGGAGGACGACACAGTG | CATACCTGGTGGGGTCTTGG | 59.8 | 452 |
| *SLC4A1* | GGTAGTTCTGTCCTTGCCCC | TCCTGATCTCGGGTGATCCA | 59.9 | 444 |
|  | AAACCTGAGCGCAAGATGGT | CCTTTCCTCCAGGATCCCCT | 60.3 | 413 |
|  | GCTCCATATGGTGCCTGTGT | GATGATCTGGATGCCCGTGA | 59.8 | 409 |
| *SPTA1* | ATGTCTTCTCCCAGGGACCC | ACCAAAGACACTGAGCACCC | 60.4 | 507 |
|  | TCTTTGAGCCAGACCAGCTG | AGCTGAGGCAAACATGAAGT | 59 | 431 |
| *EPB41* | AATCGCTTGAACCTGGGAGG | ACCAGAAACTTTCAATCCTAGGCT | 60 | 450 |
| *EPB42* | TGATGAGCCCTCCTTACCCA | GCTGCCTGGGACAACTACTT | 60 | 407 |

**Table S5** List of significant variants detected in RBC enzyme-encoding genes among patients with HS

| **Patient ID** | **Enzyme gene** | **Chromosome** | **Position** | **SNP ID** | **Variant (nucleotide)** | **Variant (amino acid)** | **Zygosity** | **ACMG classification** | **Protein gene** |
| --- | --- | --- | --- | --- | --- | --- | --- | --- | --- |
| 29 | *GSR* | Chr8 | 30537086 | rs149225584 | c.1520C>T | p.Thr507Ile | Hetero | Uncertain significance | *ANK1* |
| 30 | *ALDOB* | Chr9 | 104189903 | rs145252200 | c.401G>A | p.Arg134His | Hetero | Uncertain significance | *SPTB* |
| 56 | *GAPDH* | Chr12 | 6645884 | . | c.164A>G | p.Lys55Arg | Hetero | Uncertain significance | *SLC4A1* |

**Table S6** List of *UGT1A1* gene variants in patients with HS in Korea

| **Gene** | **Chromosome** | **Position** | **SNP ID** | **Variant (nucleotide)** | **Variant (amino acid)** | **ACMG classification** | **Patient ID** |
| --- | --- | --- | --- | --- | --- | --- | --- |
| *UGT1A1* | Chr2 | 234669144 | rs4148323 | c.211G>A | p.Gly71Arg | Pathogenic | 1,4,11,13,16,17,19,  20,21,32,33,35,38,39,  43,44,45,48,50,55,57 |
|  |  | 234669619 | rs35350960 | c.686C>A | p.Pro229Gln | Pathogenic | 22 |
|  |  | 234676872 | rs34946978 | c.1091C>T | p.Pro364Leu | Pathogenic | 24,32,46 |
|  |  | 234680955 | rs114982090 | c.1352C>T | p.Pro451Leu | Pathogenic | 19 |
|  |  | 234681059 | rs34993780 | c.1456T>G | p.Tyr486Asp | Pathogenic | 13 |

**Table S7** Clinical characteristics of patients with HS without peripheral blood spherocytes.

| **Patient ID** | **Sex/Age** | **Gene mutations** | **Family history** | **Spleno-megaly** | **Severity of HS** | **Hemoglobin (g/dL)** | **Reticulocyte count (%)** | **Total bilirubin (mg/dL)** | **OFT** | **Additional test** |
| --- | --- | --- | --- | --- | --- | --- | --- | --- | --- | --- |
| 21 | F/15yr | *SLC4A1, UGT1A1* | Sibling hemolytic anemia | - | Mo | 10.5 | 5.5 | 4.1 | NA |  |
| 35 | F/16yr | *SPTB, EPB42, UGT1A1* | - | - | Mo | 8.8 | 12.2 | 4.9 | NA | Auto- hemolysis |
| 49 | M/1yr | *SPTB, UGT1A1* | - | - | Mo | 8.0 | 3.2 | 3.3 | + |  |
| 50 | M/4yr | *ANK1* | Father, Sibling HS | - | Mo | 8.7 | 3.8 | 15.1 | + |  |
| 54 | F/8yr | - | Father HS | + | S | 7.7 | 3.4 | 1.1 | - |  |

* Abbreviation: OFT, osmotic fragility test**Table S8** Patients without RBC membrane-encoding gene mutation

| **Patient ID** | **Other**  **mutation** | **OFT** | **PB spherocytes** | **Splenectomy** | **Family**  **history of HS** | **Severity**  **of HS** | **SDS-PAGE** |
| --- | --- | --- | --- | --- | --- | --- | --- |
| **5** |  | **+** | ♦♦ | ● | AD | ▲▲▲ | Spectrin |
| **10** |  | **+** | ♦♦ | ● | (HA, mother) | ▲▲▲ |  |
| **14** |  | **+** | ♦ | ● | AD | ▲▲▲ |  |
| **17** | ***UGT1A1*** | **+** | ♦♦♦ |  |  | ▲▲ |  |
| **22** | ***UGT1A1*** | **+** | ♦ |  | AD | ▲▲▲ |  |
| **24** | ***UGT1A1*** | **+** | ♦♦ |  | (HA, mother) | ▲▲▲ |  |
| **38** | ***UGT1A1*** | **+** | ♦ |  |  | ▲ | Spectrin |
| **52** |  | **+** | ♦♦ |  |  | ▲▲ |  |
| **54** |  | - | - | ● | AD | ▲▲▲ |  |

* Abbreviation: OFT, osmotic fragility test; AD, autosomal dominant
